# A diagnosis of syphilis following a radical circumcision for suspected penile cancer

**DOI:** 10.1308/rcsann.2022.0143

**Published:** 2024-04-02

**Authors:** KH Pang, A Haider, A Freeman, P Hadway, C Bunker, A Muneer, HM Alnajjar

**Affiliations:** ^1^ University College London Hospitals NHS Foundation Trust, UK; ^2^ University College London, UK; ^3^ Division of Surgery and Interventional Science, University College London, UK

**Keywords:** Syphilis, Sexually transmitted infection, Lichen sclerosus, Penile lesion, Circumcision, Multidisciplinary

## Abstract

We present a case of a 70-year-old gentleman who was referred to our tertiary 2-week-wait penile cancer clinic with a penile mass that was ulcerated, painful and discharging. This was suspicious for penile cancer and a radical circumcision was performed to remove the diseased foreskin en bloc with the lesion that was arising from the inner foreskin. Histopathology did not reveal cancer; however, we identified spirochaetes in keeping with syphilis. This was confirmed on serology. The patient was referred to the genitourinary medicine team and treated with antibiotics. This case demonstrates a rare presentation of genital syphilis in an elderly gentleman initially referred with concerns of penile cancer. Although, rare, especially in this age group, syphilis should be considered as a differential diagnosis in a patient presenting with an ulcerated, discharging, firm penile mass, especially given that the incidence of syphilis has been rising in recent years.

## Case history

A 70-year-old Caucasian gentleman was referred to our 2-week wait tertiary penile cancer clinic with a 5-week history of worsening, blistering ulceration on the inner foreskin. The foreskin was painful, swollen and discharging purulent fluid. He received 3 days of intravenous antibiotics in the referring hospital before seeing us with minimal improvements. He had a past medical history of high blood pressure, was a non-smoker, in a stable married relationship and denied any history of sexually transmitted infections (STIs).

**Figure 1:**
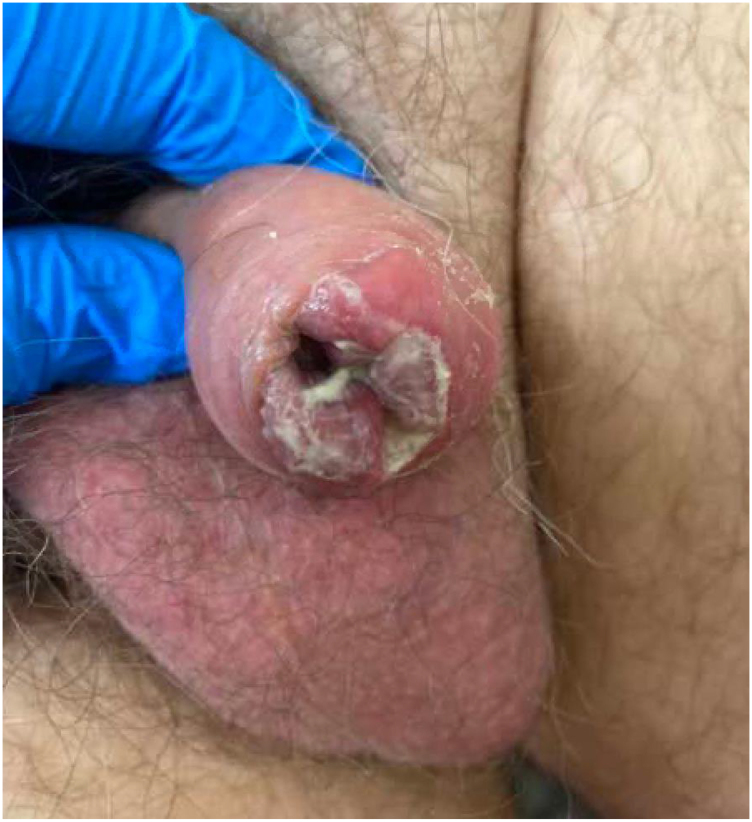
Initial presenting features. The patient had an oedematous foreskin which was discharging purulent fluid. A mass was palpable beneath the foreskin.

On physical examination, the patient had an uncircumcised penis with an oedematous foreskin and a lump palpable under the foreskin, which was tender (Figure 1). His foreskin was non retractile and that had been the case for the previous 2 weeks.

Blood tests including inflammatory markers and urine cultures were unremarkable. Computed tomography scan was unremarkable and showed no signs of primary or metastatic disease. Ultrasound groins showed no enlarged or suspicious lymph nodes.

Given the degree of evolution of symptoms in a very short period, a circumcision, with or without glans biopsy was offered. Differential diagnoses included penile cancer, an inflammatory pathology or infective bacterial or viral process.

On examination in the operating theatre, the foreskin was not retractable and the abnormal area was confined to the dorsal foreskin. Therefore, a ventral slit was performed to assess the penis. The glans and meatus were exposed, which looked normal, and the abnormality appeared to be confined to the foreskin. A radical circumcision was performed.

**Figure 2:**
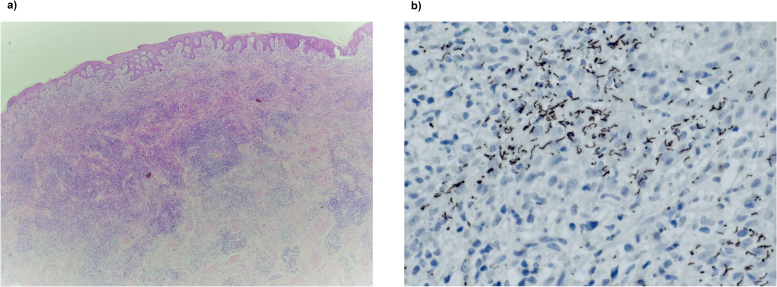
Histopathological slides. (a) Haematoxylin and Eosin stain showing a dense lymphoplasmacytic cell infiltrate in the dermis. Magnification ×10. (b) *Treponema pallidum* immunohistochemistry stain showing the presence of numerous spirochetes in the ulcerated area. Magnification ×20.

The specimen was examined in its entirety and sections show focal areas of ulceration with a dense lymphoplasmacytic and histiocytic infiltrate in the subepithelial tissue (Figure 2a). There was obliterative endarteritis and occasional clusters of histiocytic cells focally forming ill-formed noncaseating granulomas. The surface squamous epithelium showed features of lichen sclerosus (LSc), but no evidence of dysplasia, penile intraepithelial neoplasia (PeIN) or invasive malignancy. Special stains for microorganisms (periodic acid-Schiff staining with diastase (DPAS), Ziehl Neelsen, Grocott and Gram) were negative. However, immunohistochemistry for *Treponema pallidum* showed the presence of numerous coiled spirochaetes in the epidermis (Figure 2b). The features seen were in keeping with syphilis with the presence of background LSc.

**Figure 3:**
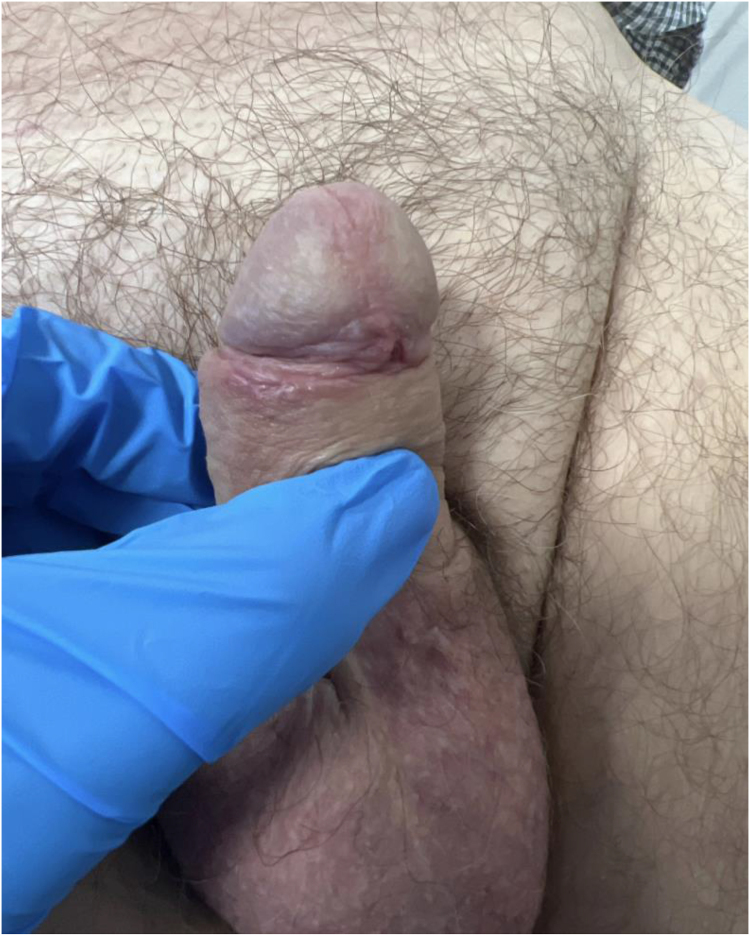
Postoperative appearance of the penis. A radical circumcision was performed and the patient’s wound had healed completely.

The patient’s microbiology screen was negative for HIV, gonorrhoea, chlamydia and mycoplasma; however, serology confirmed Treponemal antibodies. He was referred to the genitourinary medicine (GUM) team and received a course of doxycycline. The patient was seen in clinic postoperatively; his circumcision wound had healed (Figure 3), and there was evidence of a secondary rash on his chest. He claimed that the rash had much improved with doxycycline. He was also referred to the genital dermatology team for follow-up of his LSc.

## Discussion

This case demonstrates a rare presentation of a discharging penile mass. Any new penile lesions suspicious of malignancy are referred to our unit on a 2-week-wait basis. This patient presented with features of infection, was admitted for intravenous antibiotics at his local hospital, and subsequently referred to us. On examination there were features of an inflammatory/infective process and a palpable lesion. Clinically, it was concerning for penile cancer. His imaging was unremarkable and we proceeded with a radical circumcision for therapeutic and histopathological diagnosis. The pathology report was surprising in the sense that spirochaetes were seen and no features of malignancy were detected. His serology confirmed a diagnosis of syphilis.

In his initial work up, we took pus and urine swabs, and since he was married for decades and had no history of STIs, we did not perform an extensive STI screen. Given the fact that he had a palpable hard lump, surgery was inevitable to remove the lesion for treatment and diagnostic purposes.

Although the most common and concerning diagnosis of a penile mass in this age group is penile cancer, other differentials should be considered. The question is do we routinely perform less common tests such as syphilis serology testing in men presenting with a penile mass, or consider such differentials only when histopathology reveals no cancer and more common STI testing such as for gonorrhoea, chlamydia and HIV are negative. The ultimate management requires a multidisciplinary team approach, and in this case involved surgeons, radiologists, histopathologists, dermatologists and GUM specialists.

Data from Public Health England revealed a gradual increase in infectious syphilis diagnoses made in England between 2000 to 2012, with a rapid increase from 2013 (3,344 cases) to 2018 (7,541 cases). Most (75%) syphilis cases are in gay, bisexual and other men who have sex with men.^1^ Syphilis is caused by infection with the spirochaete bacterium *T. pallidum*, and is transmitted by direct contact with an infectious lesion, or in congenital cases via vertical transmission. However, only around one-third of sexual contacts of infectious syphilis will develop the disease. Following contact, the bacterium invades through mucosal surface and divides at the entry site, producing the chancre of primary disease. If untreated, around 25% of patients may develop signs of secondary syphilis (typically a rash) or, in more severe cases, tertiary syphilis, which includes neurological and cardiovascular complications. Diagnosis is from serology to detect Treponemal antibodies, polymerase chain reaction or microscopy to identify the bacterium. Management is with antibiotics in the form of penicillin or doxycycline; in addition, contact tracing is required.^2^

A recent case report also demonstrated a diagnosis of primary syphilis in a patient who presented with suspected penile cancer. Pham *et al*’s patient was much younger, aged 24 years, and presented with weight loss, inguinal lymphadenopathy and a nonulcerated penile mass. Our patient was slightly different in the sense that he was older, did not report weight loss, did have an ulcerated discharging lesion, but did not have clinical or imaging evidence of lymphadenopathy. This demonstrates that primary genital syphilis can present in varying ways. Pham *et al*’s patient also underwent a circumcision and mass excision; however, unlike our case, histopathological staining did not reveal *Treponema pallidum*, and syphilis was confirmed only on subsequent serology.^3^ In severe cases, an erosive lesion of the glans penis with associated urethrocutaneous fistulation may require a partial penectomy, as reported by Mathew *et al.*^4^

## Conclusion

A diagnosis of syphilis following a radical circumcision for suspected penile cancer is uncommon. However, this should be considered as a differential diagnosis, along with other STIs in any men presenting with acute phimosis and penile discharge since there is a rise in the incidence of syphilis. In any suspicious cases, especially in a patient with a palpable lump, staging scans and biopsies are required for definitive histopathological diagnosis.
